# Ultrasonic Testing of Mechanical Changes in a Water-Filled Pipe with Multi-Mode and Broadband Signals and Two-Level Compensation

**DOI:** 10.3390/s22228647

**Published:** 2022-11-09

**Authors:** Taeho Ju, Alp T. Findikoglu

**Affiliations:** Materials Physics and Applications (MPA), Los Alamos National Laboratory, Los Alamos, NM 87545, USA

**Keywords:** multi-mode guided waves, broadband guided waves, pipe inspection, non-destructive evaluation (NDE), structural health monitoring (SHM), environmental and operational conditions (EOC), liquid-boundary effect

## Abstract

Ultrasonic testing (UT) has been widely used for the Nondestructive Evaluation (NDE) of pipes due to its many favorable characteristics. However, one of the main challenges in the general use of UT for real-world pipelines is the sensitivity of this method to environmental and operational condition changes. This paper proposes a new UT method with enhanced compensation for environmental effects and operational condition changes. In particular, the effectiveness of the new method is tested in the presence of temperature variations, and changes in water flow rate inside a stainless-steel pipe. The proposed UT method uses multi-mode and broadband guided ultrasonic waves in the pipe walls, excited and received by single-element ultrasonic sensors that are spatially separated, forming a measurement zone between any pair of such transmit and receive sensors. Amplitude changes, time shifts, and frequency content variations in the ultrasonic signal due to temperature changes and water flow are evaluated and compensated for reliable UT of mechanical changes in the pipe. It is observed that spurious effects of water flow on ultrasonic response, if not properly compensated, can dominate over effects due to actual mechanical changes, but such liquid-boundary effects can be compensated effectively by the proposed time- and frequency-filtering method.

## 1. Introduction

Monitoring of mechanical changes due to pitting, cracking, material conversion from corrosion and/or erosion, and material addition from material migration and accumulation in pipes, especially in hard-to-access environments [1], such as under insulation or paint, is important in many industries that involve liquid or gas storage and flow [2,3]. Commercial-guided wave systems show a sensitivity to changes of 5% or above of cross-sections [4,5]. The detection of such mechanical changes under insulation is conducted most effectively by visual inspection, which is time-consuming and costly.

As an alternative solution, ultrasonic testing has been widely used in various forms such as point-by-point defect detection by bulk ultrasonic waves or long-range inspection by guided waves. Such point measurement requires a simple measurement system such as single-element transducers for transmitting and receiving ultrasonic signals while providing high precision for damage detection. The guided wave ultrasonic testing (GWUT) is more suitable for damage detection in large areas. The guided wave can propagate for a long distance along the tested structure and interrogate the whole structure in a short time, and thus it can save time and reduce the cost of the inspection.

However, these conventional UT methods have also well-known weaknesses. For example, such point-by-point inspection is time and labor-consuming and sometimes requires the shut-down of the tested facility. Thus, it entails considerable operational costs [1,6,7]. On the other hand, the conventional GWUT technique needs a complicated modal analysis of guided waves as well as a utilization of relatively complex measurement systems [4,6,8,9]. In addition, in many cases, the tested structures have non-uniform cross-sections such as bends, joints, junctions, branches, flange connections, and adapters, which can render the GWUT technique impractical.

In addition to the geometrical complexity, changes in environmental and operational conditions (EOC) can also make the implementation of GWUT difficult [10,11]. Even a straight pipe, once filled with liquid, will exhibit complex liquid-mediated ultrasonic modes and modified dispersive characteristics of guided waves compared to the case when it is empty [12,13]. In addition, such modes and dispersive characteristics are sensitively dependent on local temperature and flow conditions, thus implementing conventional GWUT challenges in the case of dynamically varying temperature and flow rate. To overcome such limitations of the conventional GWUT method mentioned above, there have been many studies in experiments and modelling of temperature variation [14,15], mechanical loads, and complex geometries [16,17].

Our earlier study proposed a multi-mode, broadband ultrasonic signal testing method for large-area structures with arbitrarily complex geometry, and experimentally demonstrated its applicability in cases with no liquid boundaries [18]. This paper shows how the technique could be modified to work satisfactorily in large-area structures with dynamic liquid boundaries, i.e., with water flow, as well as in the presence of EOC (specifically, environmental variations of temperature, and operational changes of flow rate).

To test the robustness of the new technique, this paper investigates the use of both compressive and shear transducers under stagnant, slow-flowing, and fast-flowing water conditions in a pipe. A key aspect of the new method is post signal processing on the acquired ultrasonic signals to achieve effective compensation of EOC to reveal actual mechanical changes in the structure.

The remainder of the manuscript is organized as follows: The multi-mode guided wave and underlying theory used in this study are introduced in Section 2. Section 3 shows the results of experimental studies in a pipe with different water flow rates and describes additional post signal processing for EOC compensation. Section 4 shows updated results after applying the new method to the acquired data. Section 5 summarizes the experimental and analytical results.

## 2. Theory

### 2.1. Multi-Mode Ultrasonic Signals by Non-Axisymmetric Partial Loading

**Figure 1:**
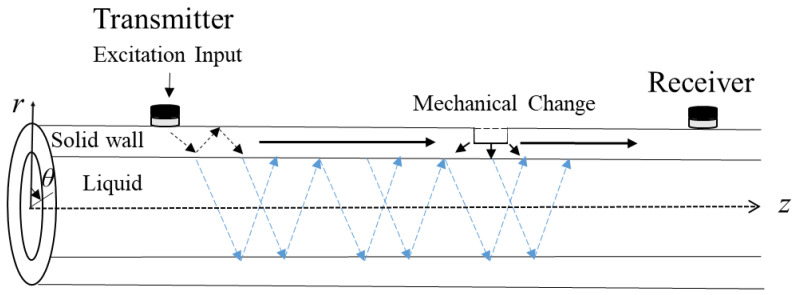
Schematic illustration of the multi-mode guided and liquid-mediated waves propagating in a liquid-filled pipe.

Non-axisymmetric partial loading by a small single-element transducer excites multiple modes (both longitudinal and flexural guided waves) in a pipe [19]. The excited flexural modes have displacement fields in all three directions (radial, circumferential, and axial). When the excitation source input is made with a wide range of frequencies, many more flexural guided wave modes are generated, and their diverse wavelengths are beneficial to have strong interactions with defects of various sizes in the entire interrogated structure. To utilize multiple frequencies, i.e., broadband operation, this study uses a linear chirp signal as an input. The input source with a linear chirp is expressed as a displacement in the radial or circumferential direction as

(1) $$u_{in}(t)=A\sin[2\pi f_{\min}t+\pi (f_{\max}-f_{\min})t^{2}/T_{d}]$$

where *f*_min_ and *f*_max_ are the minimum and maximum frequencies, respectively, *T_d_* is the duration of the chirp, and *A* is the initial amplitude of the chirp. Once external force is loaded on a rigid pipe, multiple guided modes are generated, having displacements in the *r*, *θ*, and *z* directions in the cylindrical co-ordinate, as seen in Figure 1.

According to Ref. [20], a partial loading excites a range of vibrating modes in different orders. Since each mode has different wavelengths and displacement profiles, multi-mode, broadband ultrasonic signals used in this study can interact with and detect mechanical changes in different sizes and shapes. Furthermore, the use of multi-mode and broadband signals allows good measurement sensitivity in the presence of geometric non-uniformities.

### 2.2. Baseline and Monitoring Ultrasonic Signals

Figure 1 shows a schematic illustration of the propagation of ultrasonic signals in a pipe filled with liquid. The propagating ultrasonic signals are comprised of guided waves in the solid wall as well as liquid-mediated modes in the liquid. The guided ultrasonic signals are then scattered, mode converted, or trapped by mechanical defects, such as geometrical discontinuities, cracks, wall losses, material deposits, etc.

In the proposed method, first a baseline transmission signal, *T_b_(t)*, is obtained in the interrogated zone, where no mechanical change is present. Then a monitoring transmission signal, *T_m_(t)*, is obtained in the same interrogated zone to monitor any mechanical changes. The baseline and monitoring signals should be adjusted and normalized to achieve long-term reliability of the proposed technique in case of changing measurement conditions, e.g., due to potential degradation of sensors or coupling medium. For this purpose, this paper takes the following steps: (1) remove DC components, *(T)_avg_*, from the baseline transmission signals and the monitoring transmission signals, where *(T)_avg_* is a single scalar quantity calculated as the average of all digitized amplitudes in the time domain; i.e., implement Equations (2) and (3) to remove the biased offset in the amplitude.

(2) $$x_{b}(t)=T_{b}(t)-{\left(T_{b}\right)}_{avg}$$

(3) $$x_{m}(t)=T_{m}(t)-{\left(T_{m}\right)}_{avg}$$

(2) normalize the DC offset-removed time domain signals, $x_{b,m}(t)$ by the positive maximum amplitude, $x_{\max}$ as

(4) $${\tilde{x}}_{b}(t)=x_{b}(t)/{\left(x_{b}\right)}_{\max}$$

(5) $${\tilde{x}}_{m}(t)=x_{m}(t)/{\left(x_{m}\right)}_{\max}$$

### 2.3. 1st EOC Compensation Method

If the interrogated structure is filled with liquid, the environmental and operational condition (EOC) changes can have a significant effect on the acquired signals, potentially masking the “mechanical change” information. In other words, when EOC changes occur, the propagating medium and corresponding ultrasonic wave behavior also change, limiting the effectiveness of the baseline comparison in distinguishing defect formation or damage in the structure from spurious effects due to EOC changes.

Spurious effects due to EOC changes, such as due to temperature variations, are most commonly seen in the form of phase-shifts in the acquired transmitted signals. To remove such EOC effects, this study first divides both the baseline and monitoring signals into several bins in the time-domain [18,21]. Then, the cross-correlation function is applied to each bin of the baseline and monitoring signals to calculate the time delay for maximum coherence to achieve effective phase-shift compensation. The details of the phase-shift compensation method for empty pipes have been reported previously [15].

The cross-correlation in each bin can be written as

(6) $$R^{i}(t+\tau )=\frac{1}{2T}\int_{t}^{t+T}{\tilde{x}}_{b}(s){\tilde{x}}_{m}(s+\tau )ds,$$

where the superscript *i* indicates *i*-th bin, *T* is the length of the bin, and *τ* is a time delay offset. The monitoring signal is rearranged in the new time domain in a way to maximize the coherence and minimize the difference. Note, that this process compensates for the phase-shift effect due to EOC, while mostly leaving effects in the signal due to mechanical changes intact, as was reported previously [15] in the case of empty pipes.

### 2.4. 2nd EOC Compensation Method

**Figure 2:**
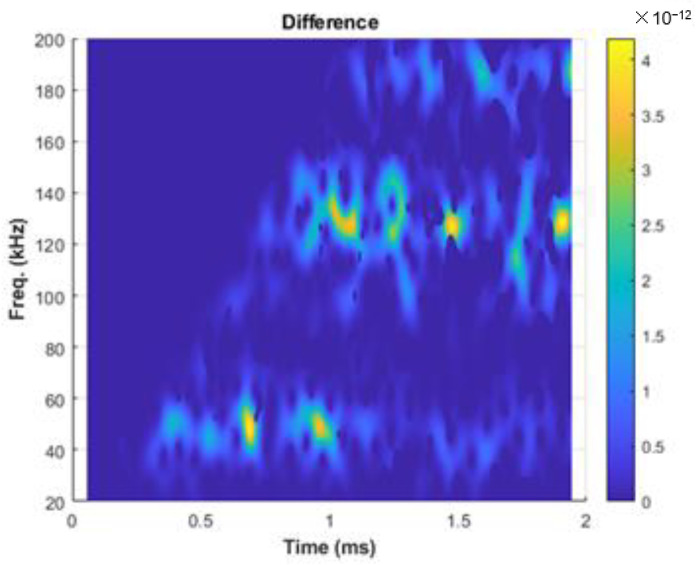
Example of the Short-Time Fourier Transform (STFT) of the difference signal, shown as a 2D contour map in the frequency–time domain.

For the 2nd EOC compensation method, a difference transmission signal is defined as a difference between the normalized monitoring transmission signal, ${\tilde{x}}_{m}(t)$, and the normalized baseline transmission signal, ${\tilde{x}}_{b}(t)$. Next, Short-Time Fourier Transforms (STFT) [22] of the difference signals is performed to reveal mixed time-frequency characteristics of the difference signal, as shown in Figure 2.

Although a large portion of spurious effects due to EOC changes can be compensated using the 1st compensation method, our experience has been that liquid-boundary effects due to the presence of liquid in the pipe render the 1st compensation method insufficient for full compensation. Thus, this paper proposes a second, additional, compensation method as a further improvement.

If the difference signal is obtained in a relatively short time when there is no mechanical change that has occurred or been made and there is no significant temperature variation during that time, any non-zero power intensity in STFT can be attributed to EOC changes that are dominated by liquid-boundary effects (due to the presence of water).

**Figure 3:**
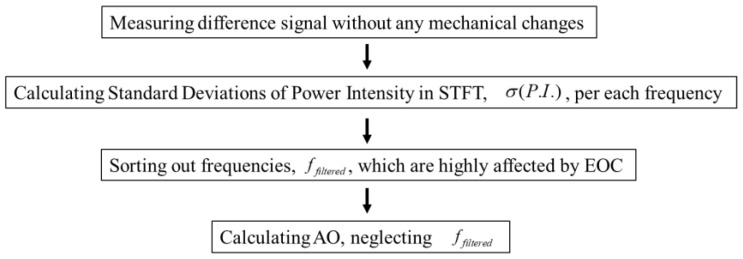
Flow chart for the implementation of the 2nd EOC compensation method.

**Figure 4:**
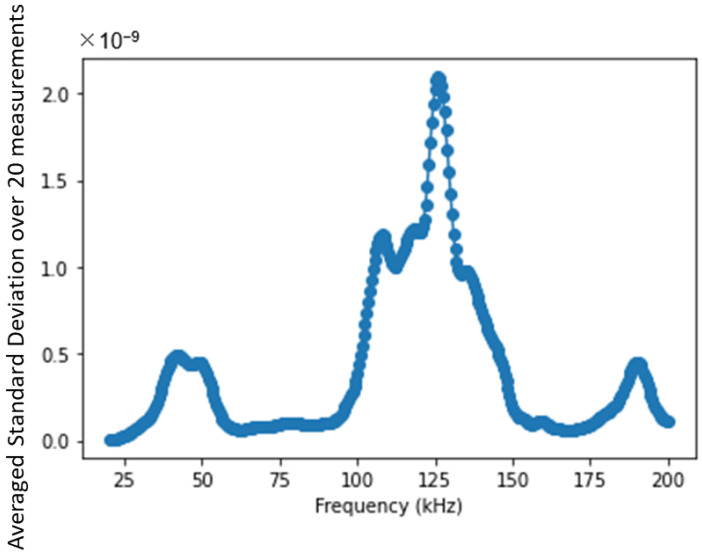
Averaged standard deviation of power intensity at each frequency over 20 measurements.

Based on this observation and assumption, the 2nd EOC compensation method is implemented as shown diagrammatically in Figure 3. First, a baseline and a monitoring signal are acquired within the shortest time interval possible (in practice, <1 min is easily achievable), from which a difference signal is calculated. The difference signal thus obtained will be assumed to be predominantly due to liquid-boundary effects with no significant contribution from temperature variation or mechanical changes in the interrogated structure. Then, the difference signal is mapped in the frequency–time domain, using STFT, and the standard deviations of power intensity in the frequency–time domain are calculated per each frequency. This procedure is repeated multiple times till convergence is achieved with a good signal-to-noise ratio in the spectral content. Figure 4 shows an example of the averaged standard deviations of power intensity in STFT for frequencies from 20 to 200 kHz. This profile provides critical information as to which frequencies are most sensitive to the presence of liquid than others. The data corresponding to the most sensitive frequencies are excluded from the estimation of mechanical changes so that the remaining frequency bands will be less dominated by liquid-boundary effects. The degree of exclusion will depend on the specifics of the interrogated zone. This paper shows that even in the case of exclusion of data corresponding to up to 95% of the frequencies, the least sensitive data, related to the remaining 5% of the frequencies, still provides a sufficient signal-to-noise ratio for monitoring of small (<1% localized volume change) mechanical change in the interrogated zone of pipes with fast-flowing water inside.

As a simple measure of mechanical changes, a scalar quantity, Actionable Output (*AO*), is introduced in this paper, which is defined as

(7) $$AO=\frac{[\sum_{k=1}^{F}\sigma (f_{k})]}{F}$$

where *F* is the number of least sensitive frequencies used and $\sigma (f_{k})$ is a standard deviation of power intensity in STFT at each frequency *f_k_*.

In the following sections, the correlation of *AO* with mechanical changes will be investigated, i.e., the effectiveness of the proposed two-level compensation method, in a pipe at different water-filling conditions and different water flow rates.

## 3. Experimental Setup

**Figure 5:**
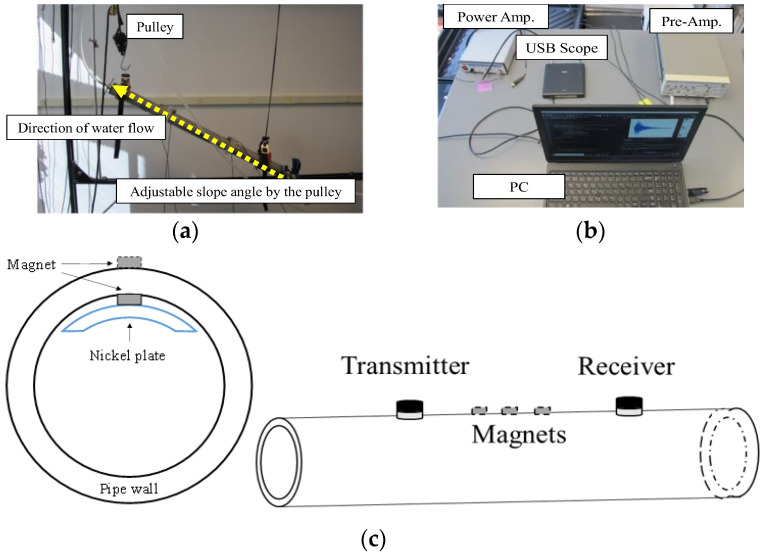
Experimental setup for multi-mode ultrasonic signal measurement using adjustable tilt angle (**a**); electronics for multi-mode ultrasonic measurements (**b**); and cross-section of stainless- steel pipe (**c**): Nickel plate is used as a holder of inner magnets. The magnets drawn with a solid line stay on the inner surface of the pipe, keeping their initial locations. The magnets shown with a dashed line are attached on the outer surface to simulate mechanical changes.

In this section, it is presented in detail how the proposed UT method with a two-level EOC compensation scheme can detect mechanical changes in a pipe with dynamic liquid-boundary conditions. For the test, a pair of longitudinal or shear piezoelectric ultrasonic transducers (V103-RM for longitudinal and V153-RM for shear, Panametrics^TM^, Waltham, MA, USA) are attached on a 0.91 m (3 ft) long, 50.8 mm (2″) sch. 40 stainless steel pipe having a 0.3 m (1 ft) long separation between the transmitter and receiver transducers. This pipe is connected to a pulley to adjust the tilt angle, which allows different water-filling conditions to be achieved in the pipe, as shown in Figure 5a. The interrogation zone is defined as the area between the receive and transmit transducers. Signal generation and acquisition are made using a portable function generator and scope (Handyscope HS5, TiePie, Sneek, The Netherlands), and controlled using a Python script on a laptop computer, see Figure 5b. To achieve a high signal-to-noise ratio (SNR), a power amplifier (WMA-100, Falco Systems, Merida, Mexico) is used on the transmit side, and a pre-amplifier (SR 560, Stanford Research System, Sunnyvale, CA, USA) is used on the receiver side. An averaging factor of 800 is used for each signal acquisition. A submersible miniature pump (12 Volt Stainless Steel Mini Monsoon^®^XL 60 Pump, Proactive Environmental Products, Bradenton, FL, USA) is used to achieve an adjustable water-flow rate in the pipe.

In this study, three square plate magnets (NSN0610, MAGCRAFT^®^ Advanced Magnetic Materials, Canton, MI, USA) with dimensions of 12.7 mm (1/2 in.) × 12.7 mm (1/2 in.) × 3.175 mm (1/8 in.) are attached to simulate mechanical changes on the stainless-steel pipe. To allow for magnet perturbation studies on the non-magnetic SS pipes, other magnets and nickel plates are placed permanently inside the pipe prior to outer magnet attachment, as shown in Figure 5c.

This experimental setup allows tests to be conducted under different water flow rates and pipe-filling conditions: stagnant water, slow water flow (17 mL/s), and fast water flow (85 mL/s), with half-filled and fully filled pipe conditions. In the following section, the proposed compensation methods are tested under different EOC.

## 4. Estimation of Mechanical Changes in the Presence of EOC Changes

### 4.1. Empty Pipe

For the first measurement, the pipe is used empty, i.e., without liquid boundary. Either a pair of compressive or shear transducers are used for compressional or shear excitation and detection, respectively. The experimental results from both the compressional (radial) and shear (circumferential) excitations are included in this study to investigate the effect of proposed EOC compensation on the different wave fields. In particular, the shear excitation is expected to have less interference from the liquid boundary due to minimal acoustic coupling between the pipe wall and the liquid inside the pipe for dominant shear modes that are excited by shear excitation. Three magnet perturbations are made within the interrogated zone, defined as the pipe section between the transmit and receive transducers. A linear chirp signal from 20 to 200 kHz with 1 ms duration is used as excitation, and the response is acquired for 2 ms with an appropriate delay corresponding to the fastest signal component arrival. The proposed EOC compensation method is not applied for the initial test.

**Figure 6:**
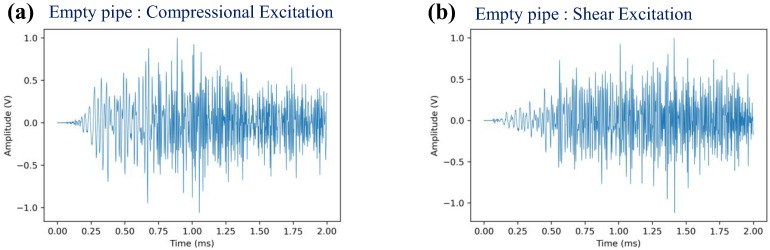
Time domain signals for the empty pipe with compressional (**a**) and shear (**b**) excitations.

**Figure 7:**
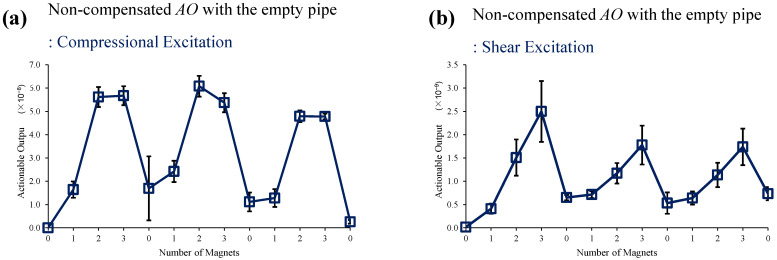
Changes in *AO* with the different number of magnet perturbations: the empty pipe with (**a**) compressional and (**b**) shear excitations, where no EOC compensation method is applied.

Figure 6 shows the multi-mode transmission signals which are used for *AO* estimation. Figure 7 shows the test result of magnet perturbations on the empty pipe. One baseline and thirteen monitoring signals are measured for one complete set. The cyclic magnet perturbations on the outer surface (0, 1, 2, 3 magnets) are shown in Figure 5, and 0 magnet indicates no magnet was attached. It repeated three times, i.e., each *AO* and error bar are calculated from three consecutive measurements. As mentioned above, without EOC compensation, the detection sensitivity of the method is poor. It is seen that overall, the *AO* has a large experimental error and includes drift in the no-magnet perturbation condition. Note that this short segment of pipe has resonance modes due to multiple reflections from its ends, and to avoid their deleterious effect of such boundary conditions on the perturbation studies, the signals were filtered in time and frequency domains so that only the times and frequencies of interest in the STFT map are used for the analysis.

**Figure 8:**
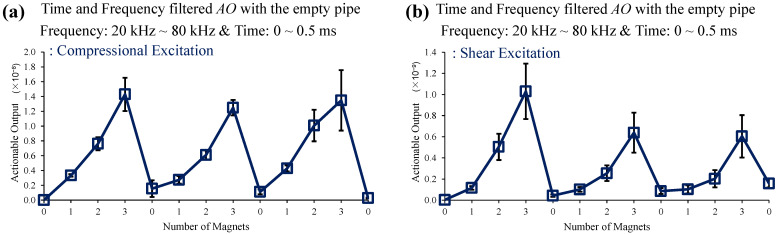
Changes in *AO* with the different number of magnet perturbations: the empty pipe with (**a**) compressional and (**b**) shear excitations. Simple time and frequency filtering lead to improved detection.

Figure 8 shows an example of such time and frequency filtering, improving the *AO* dependence on magnet perturbation. It is seen that such simple filtering can improve measurement accuracy.

**Figure 9:**
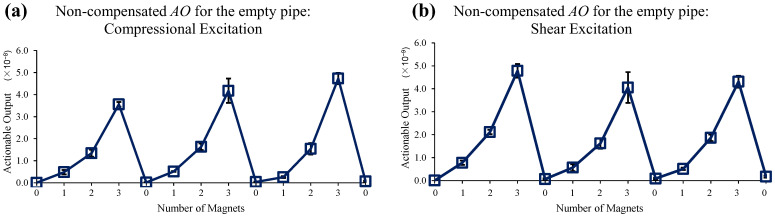
Changes in *AO* with the different numbers of magnet perturbations for the empty pipe with (**a**) compressional and (**b**) shear excitations. No EOC compensation is applied.

Based on this positive result, similar time and frequency filtering are used for the measurements reported in the rest of the paper. Figure 9 shows that *AO* with the empty pipe can track the magnet perturbation effect with high accuracy without the need for two-level EOC compensation. In addition, in this empty pipe case, both compressional and shear excitations provide similar sensitivity to the given mechanical changes.

### 4.2. Stagnant Water

**Figure 10:**
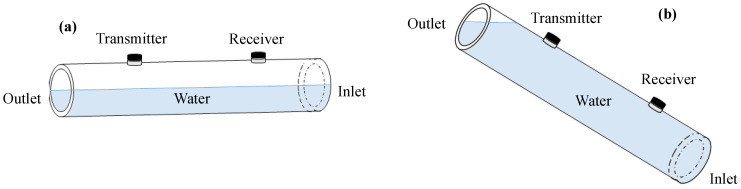
Schematic representation of (**a**) half-filled pipe and (**b**) fully filled pipe.

As the first measurement with liquid-boundary conditions, the pipe is filled with stagnant water. Either a pair of compressive or shear transducers are used for compressional or shear excitation and detection, respectively. In addition, two different tilt angles are used to achieve half-filled and fully filled pipe conditions, as shown in Figure 10.

**Figure 11:**
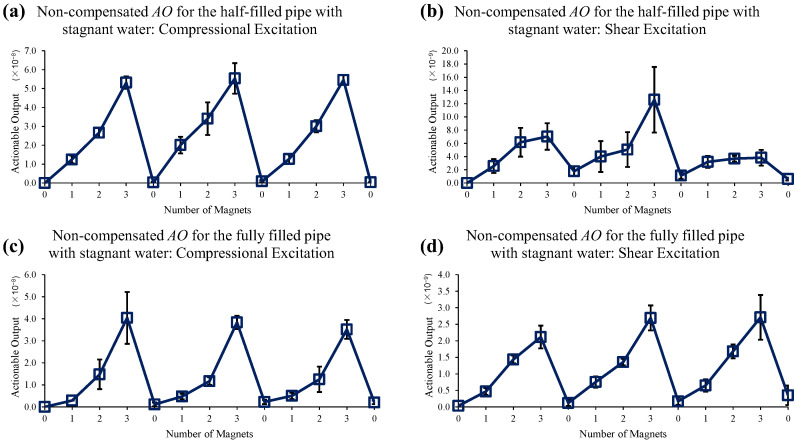
Changes in *AO* with the different number of magnet perturbations for the half-filled pipe with (**a**) compressional and (**b**) shear excitations, and for the fully filled pipe with (**c**) compressional and (**d**) shear excitations. No EOC compensation method is applied.

For this test, a linear chirp signal from 20 to 100 kHz with a 0.5 ms duration is used, and the response is acquired with a 1.2 ms time window. Figure 11 shows the test result of magnet perturbations on the half-filled and fully filled pipes with stagnant water without using the EOC compensation method. It is seen that the *AO* with the compressional excitation shows a good sensitivity to the magnet perturbations. However, the measurements with the shear excitation for the half-filled pipe have a large experimental error and include a relatively high level of spurious signals. These measurements indicate that even the static liquid boundary, due to stagnant water, might necessitate the implementation of EOC compensation methods to use the right time and frequency filtering and phase-correction, which is realized by the proposed compensation methods.

**Figure 12:**
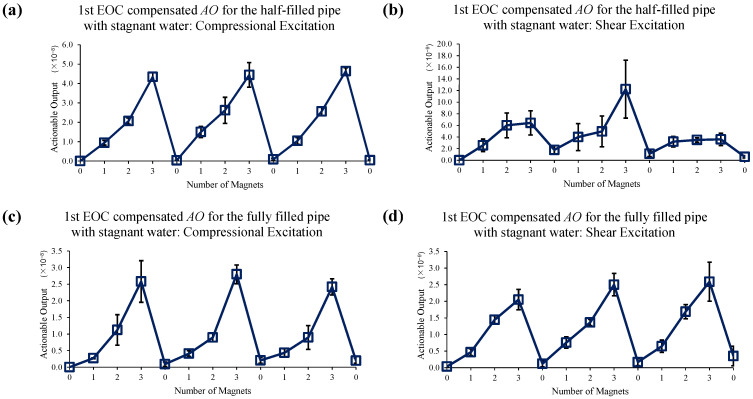
Changes in *AO* with the different number of magnet perturbations for the half-filled pipe with (**a**) compressional and (**b**) shear excitations; and the fully filled pipe with (**c**) compressional and (**d**) shear excitations. In this case, only the 1st EOC compensation method is applied.

Figure 12 shows the effect of the implementation of the 1st EOC compensation method. It is observed that a purely phase-correction compensation method is not sufficient to improve the measurement accuracy (especially in the case of shear excitation in a half-filled pipe) when a liquid boundary exists.

**Figure 13:**
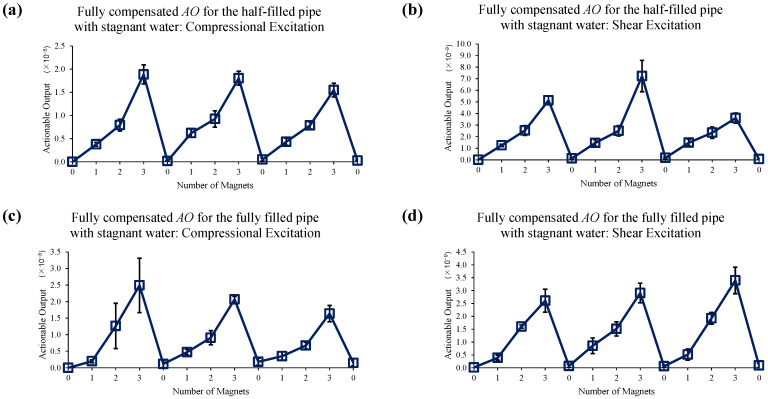
Changes in *AO* with the different number of magnet perturbations: the half-filled pipe with compressional and shear excitations (**a**,**b**), and the fully filled pipe for the two excitation modes (**c**,**d**). The full EOC compensation method is applied.

Figure 13 shows the test results when the full, i.e., two-level, EOC compensation method is applied. Under all investigated conditions, the *AO* tracks the changes in the number of magnet perturbations. One can conclude that the accuracy and sensitivity of the proposed UT method are significantly improved once it is combined with the proposed two-level compensation. In the next section, it is investigated whether such accuracy and sensitivity improvement are preserved in the case of dynamic liquid-boundary conditions, i.e., with water flow.

### 4.3. Water Flow

In this section, two water-flow conditions are investigated: slow water flow (17 mL/s), which is expected to be mostly laminar; and fast water flow (85 mL/s), which might include some turbulent flow at the inlet of the pipe. In this section, the same magnet perturbation tests of the previous section are repeated, and all other experimental conditions are the same, while introducing different water-flow conditions.

**Figure 14:**
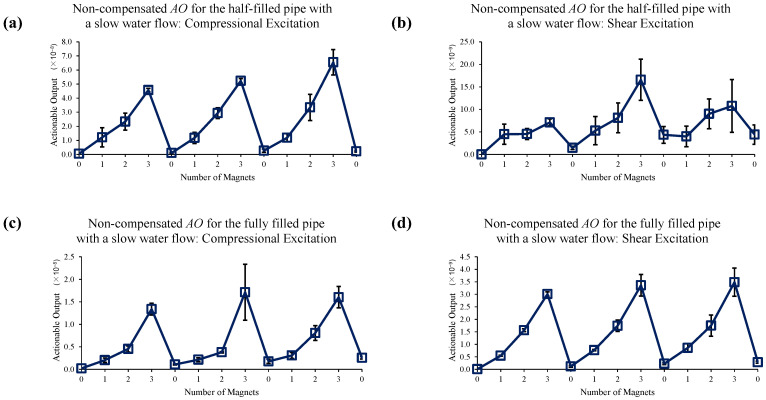
Changes in *AO* under slow water-flow conditions with the different number of magnet perturbations for the half-filled pipe with (**a**) compressional and (**b**) shear excitations; and the fully filled pipe with (**c**) compressional and (**d**) shear excitations. No EOC compensation method is applied.

**Figure 15:**
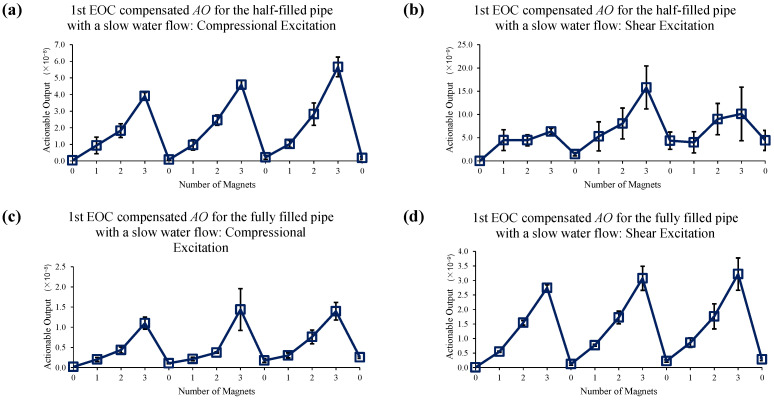
Changes in *AO* under slow water-flow conditions with the different number of magnet perturbations for the half-filled pipe with (**a**) compressional and (**b**) shear excitations; and the fully filled pipe with (**c**) compressional and (**d**) shear excitations. Only the 1st EOC compensation method is applied.

**Figure 16:**
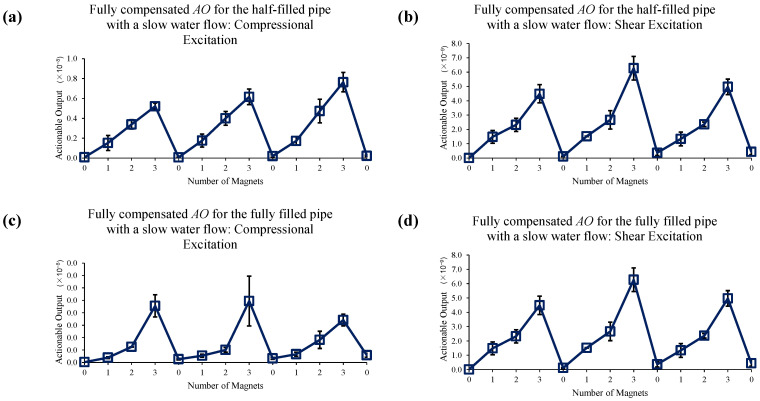
Changes in *AO* under slow water-flow conditions with the different number of magnet perturbations for the half-filled pipe with (**a**) compressional and (**b**) shear excitations; and the fully filled pipe with (**c**) compressional and (**d**) shear excitations. The full, two-level EOC compensation method is applied.

Figure 14 shows the reference data set for slow water flow case when no EOC compensation is applied. Sole application of 1st EOC compensation method does not improve detection sensitivity or accuracy, as seen in Figure 15. However, Figure 16 shows application of full EOC compensation method leads to significant improvement in detection sensitivity and accuracy, especially in the case of half-filled pipe with shear excitation.

**Figure 17:**
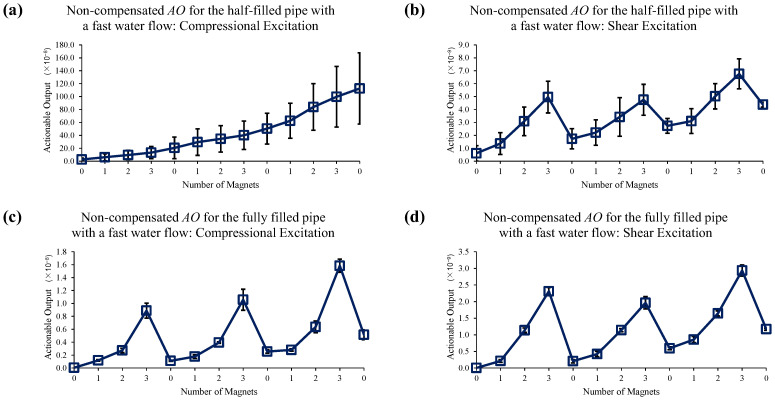
Changes in *AO* under fast water-flow conditions with the different number of magnet perturbations for the half-filled pipe with (**a**) compressional and (**b**) shear excitations; and the fully filled pipe with (**c**) compressional and (**d**) shear excitations. No EOC compensation method is applied.

**Figure 18:**
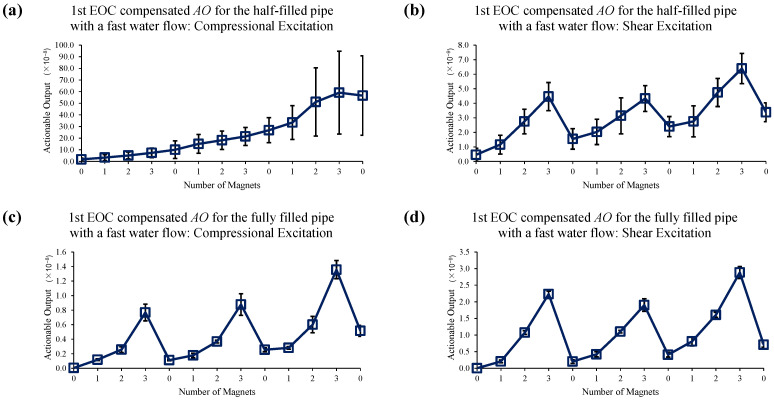
Changes in *AO* under fast water-flow conditions with the different number of magnet perturbations for the half-filled pipe with (**a**) compressional and (**b**) shear excitations; and the fully filled pipe with (**c**) compressional and (**d**) shear excitations. Only the 1st EOC compensation method is applied.

**Figure 19:**
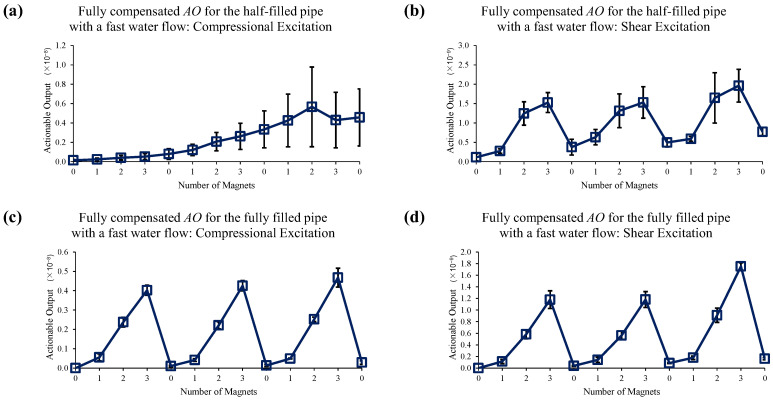
Changes in *AO* under fast water-flow conditions with the different number of magnet perturbations for the half-filled pipe with (**a**) compressional and (**b**) shear excitations; and the fully filled pipe with (**c**) compressional and (**d**) shear excitations. The full, two-level EOC compensation method is applied.

Figure 17 shows the reference data set for the fast water flow case when no EOC compensation is applied. In this case, detection sensitivity is significantly degraded overall, but especially under half-filled pipe conditions. The sole application of 1st EOC compensation method does not improve detection sensitivity or accuracy in any significant manner, as seen in Figure 18. The application of full EOC compensation leads to appreciable improvement in all cases, as seen in Figure 19. However, Figure 19 also shows the limitation of the proposed compensation method, especially in the most challenging case of half-filled pipes with a compressional excitation under fast flow conditions. When the pipe is half-filled, there is more space for water to fluctuate within the pipe. This phenomenon of liquid-boundary fluctuations can be intensified by the fast water flow. Therefore, the fast water flow in the half-filled pipe leads to the most challenging conditions for EOC compensation for both excitations. Nevertheless, since the shear excitation has a dominant displacement field in the circumferential direction and has minimal coupling to the liquid inside, the proposed EOC compensation still performs satisfactorily in that case.

## 5. Summary and Conclusions

In this manuscript, a multi-mode and broadband ultrasonic testing technique is described for non-destructive large-area inspection and monitoring of mechanical changes in pipes with static and dynamic liquid boundaries. The technique uses two single-element sensors either for compressional or shear ultrasonic excitation for transmission type measurement. The difference transmission signal to a prior baseline measurement is analyzed to determine *AO*, a scalar quantity, which is a measure of mechanical changes. To assess the feasibility of the proposed technique in the case of pipes with liquid boundaries, a pipe system with adjustable tilt angle (half- and fully filled) and flow rate (stagnant, slow, fast) is constructed. The experimental results show that both the compressional and shear excitation modes provide a similar level of accuracy and sensitivity to the magnet perturbations once the UT method is improved with the two-level EOC compensation, which appear to be adequate for stagnant and slow flow cases under both partially and fully filled pipes. Under fast flow conditions, the performance of the technique degraded significantly, especially for the half-filled pipe case.

## Data Availability

Not applicable.
